# Comparing parecoxib and ketorolac as preemptive analgesia in patients undergoing posterior lumbar spinal fusion: a prospective randomized double-blinded placebo-controlled trial

**DOI:** 10.1186/s12891-015-0522-5

**Published:** 2015-03-18

**Authors:** Koopong Siribumrungwong, Julin Cheewakidakarn, Boonsin Tangtrakulwanich, Sasikaan Nimmaanrat

**Affiliations:** Department of Orthopedic Surgery and Physical Medicine, Faculty of Medicine, Prince of Songkla University, Songkla, Hat Yai 90110 Thailand; Department of Anesthesiology, Faculty of Medicine, Prince of Songkla University, Songkla, Hat Yai 90110 Thailand

**Keywords:** Parecoxib, Ketorolac, Preemptive analgesia, Posterior lumbar fusion

## Abstract

**Background:**

Poor postoperative pain control is frequently associated with complications and delayed discharge from a hospital. Preemptive analgesia is one of the methods suggested for reducing postoperative pain. Opioids are effective for pain control, but there known addictive properties make physicians cautious about using them. Parecoxib and ketorolac are potent non-opioid NSAIDs that are attractive alternative drugs to opioids to avoid opioid-related side effects. However, there are no good head-to-head comparisons between these two drugs in the aspect of preemptive analgesic effects in lumbar spinal fusion surgery. This study aimed to compare the efficacy in terms of postoperative pain control and safety of parecoxib with ketorolac as preemptive analgesia in posterior lumbar spinal fusion patients.

**Methods:**

A prospective, double-blinded randomized controlled trial was carried out in patients undergoing posterior lumbar spinal fusion, who were randomized into 3 groups (n = 32). Parecoxib, ketorolac or a placebo was given to each patient via injection around 30 minutes prior to incision. The efficacy of postoperative pain control was assessed by a verbal numerical rating score (0–10). And various postoperative things were monitored for analysis, such as total opioid consumption, complications, and estimated blood loss.

**Results:**

Both the ketorolac and parecoxib groups showed significantly better early postoperative pain reduction at the postanesthesia care unit (PACU) than the control group (*p* < 0.05). There were no differences between the pain scores of ketorolac and parecoxib at any time points. Complications and bleeding were not significantly different between all three groups.

**Conclusions:**

Preemptive analgesia using both ketorolac and parecoxib showed a significantly better early postoperative pain control in the PACU than the control group in patients undergoing lumbar spinal fusion.

**Trial registration:**

ClinicalTrials.gov NCT01859585. Registered 15 May 2013.

## Background

Inappropriate postoperative pain control has been associated with a number of complications such as delay in discharge from the hospital, atelectasis, pulmonary edema, hypoxemia, and cardiovascular system complications. It can also delay early mobilization and increase the risk of thromboembolism, and lead to reduced bladder and intestinal motility [1,2]. Appropriate postoperative pain control is associated with lower rates of morbidity and mortality and also shorter hospitalization which reduces overall costs [3].

Opioid therapy is recommended as the first choice medication for postoperative pain control but is associated with a number of adverse effects [4]. Multimodal or balanced analgesia using a combination of analgesic methods throughout the preoperative and postoperative periods to control postoperative pain is recommended over the use of opioids alone [4,5]. Nonsteroidal anti-inflammatory drugs (NSAIDs) are one of the options being trialed in order to reduce postoperative pain and avoid the adverse effects of opioids [6,7]. These drugs provide a potent analgesic effect with a lack of sedative and opioid side effects. NSAIDs have effective opioid-sparing analgesic effects while reducing morphine consumption up to 27% in the first postoperative 24 hours [8].

Ketorolac (Toradol, Hoffman-La Roche Inc., Nutley, NJ) is an injectable NSAID with strong analgesic activity. Ketorolac is an established non-selective NSAID administered in an active form [9-11]. The efficacy of ketorolac in decreasing postoperative pain following orthopedic surgical procedures including spine surgery has been previously demonstrated [12-16].

Parecoxib is a specific inhibitor of cyclo-oxygenase2 enzymes (COX-2). Parecoxib is metabolized by the human liver to produce valdecoxib which produces analgesic effects. Many studies have shown the efficacy of parecoxib in reducing postoperative pain [17-21] while not affecting platelet aggregation [22]. Parecoxib has been used in many kinds of surgery for postoperative pain control [23-31].

Postoperative pain following lumbar spinal fusion surgery can be perceived by the patient immediately in the recovery room as they are recovering after general anesthesia. Pain management in the postanesthesia care unit (PACU) is very important and better management of pain in the PACU setting would likely improve patient satisfaction and facilitate shorter PACU stays.

The efficacy of using NSAIDs as preemptive analgesia is still controversial [32]. Both Ketorolac and Parecoxib are available in injectable forms and have been shown to have the ability to reduce postoperative pain given either before or after surgery. Both are potent non-opioid, nonsteroidal anti-inflammatory drugs. They are of interest to pain control physicians, as their use avoids many opioid-related side effects while providing good analgesia. However, there are no good head-to-head comparisons between these two drugs addressing their preemptive analgesic effects in lumbar spinal fusion surgery. The purpose of this study was to compare the efficacy of preemptive parecoxib and ketorolac as postoperative pain control agents, especially in the PACU in patients who have undergone posterior lumbar spinal fusion. We hypothesized that a single dose of preemptive analgesia using either parecoxib or ketorolac could reduce early postoperative pain compared to the placebo group.

## Methods

### Study population

**Figure 1:**
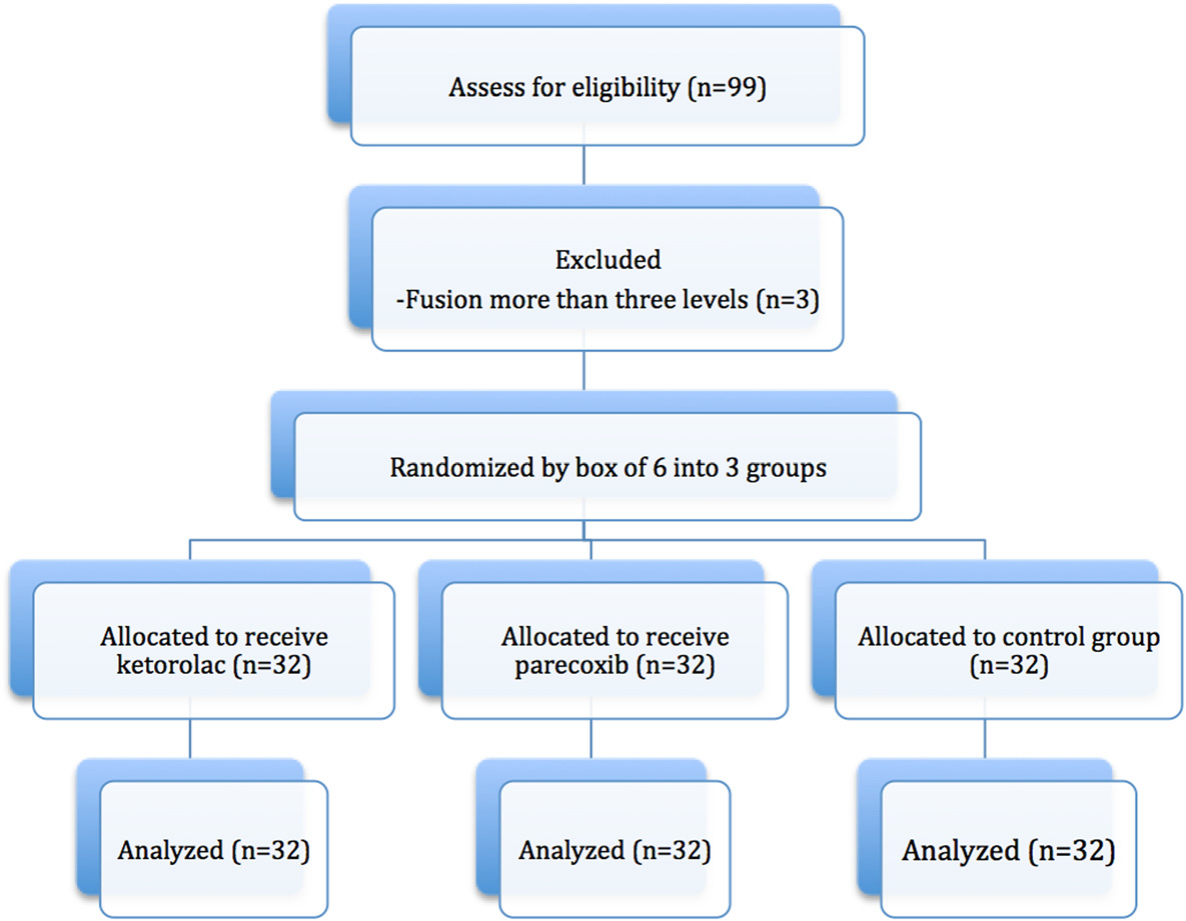
**Flow diagram of the study.**

This was a prospective double-blinded randomized controlled trial. This study was approved by the Ethics Committee at the Faculty of Medicine, Prince of Songkla University. The trial was conducted in accordance with the Helsinki II declaration. The Consolidated Standards of Reporting Trials (CONSORT) recommendations for reporting randomized controlled clinical trials were followed [33] (Figure 1). All patients signed a written informed consent form before surgery. Ninety-nine consecutive patients were enrolled in this study between March 2011 and July 2013. Inclusion criteria were patients who were diagnosed as lumbar disc herniation, spondylolisthesis, spinal stenosis, and had indications for decompressive laminectomy and fusion for one to three levels. Eligible patients were aged 18–80 years and had an American Society of Anesthesiologist physical status (ASA) classification of I-II. Exclusion criteria were a history of NSAIDs or opioid or sulfonamide allergy, any coagulopathy disease or patients who current use of antiplatelet or anticoagulant drugs, severe hepatic impairment, acute peptic ulceration, congestive heart failure, pregnancy, and lactation.

### Randomization, allocation concealment and blinding procedures

All patients were randomly allocated to receive parecoxib or ketorolac or placebo by block of 6 randomization using a computer generated random number (http://www.randomizer.org/). The sequential random number code was enclosed in a sealed opaque envelope to ensure allocation concealment. To ensure blinding, the nursing personnel were not involved in evaluation of the patients. The code was kept confidential until the time of data analysis. All patients, data collectors, and health care providers involved in postoperative management were blinded to the group allocation.

### Intervention

The parecoxib group received 40 mg of parecoxib intravenously, the ketorolac group received 30 mg of ketorolac intravenously, and the placebo group received 10 mL of saline intravenously. All patients received their medication 30 minutes before surgery from the anesthesiologist. All patients were admitted one day before surgery in order to prepare them and give them instructions how to assess their pain using a verbal numerical rating score (VNRS) from 0 (no pain) to 10 (worst imaginable pain).

The anesthetic technique was standardized among the participating anesthesiologists. The same anesthetic protocol for preoperative medication, induction, and maintenance was used by all anesthesiologists. Anesthesia was induced with propofol (2 mg/kg) and fentanyl (2 microgram/kg). Orotracheal intubation was facilitated with vecuronium (0.15 mg/kg). Nitrous oxide-oxygen-narcotic anesthetic was used for all surgical procedures with inhalation agents used as adjuncts to control blood pressure. There was no local anesthesia usage before or after surgery. The surgical technique was also standardized among the participating surgeons, who performed a decompressive laminectomy with posterolateral fusion (one to three levels) with pedicular screws fixation. Local bone graft plus hydroxyapatite and tricalcium phosphate composite (Triosite biomatlante Sarl, Vigneux de Bretagne, France) were used for spinal fusion. Operating microscopes were not used. All patients received the same postoperative pain management, consisting of paracetamol (500 mg) and intravenous morphine for rescue postoperative pain control. No other analgesic supplement was given during the study period.

### Data collection and assessment

The basic characteristics of the patients, including age, gender, height, weight, type of surgery, including variables such as the number of levels, duration, intraoperative blood loss, and amount of postoperative bleeding, were recorded. After surgery, patients were requested to rate their pain intensity using the VNRS. When the patients regained consciousness from general anesthesia after surgery the study procedure was recorded as time 0. The VNRS was recorded at 0, 1, 2, 3, 4, 6, 12, 18 and 24 hours after surgery by data collectors. The amounts of intravenous morphine requested were also recorded. Side effects of the analgesic procedures were also recorded, such as dyspepsia, nausea/vomiting, constipation, dizziness, respiration depression (respiratory rate less than 8 breaths per minute), and pruritus. The amount of drain output was recorded until removal.

### Statistical analysis

We used Shapiro-Wilk test to check the distribution of the data. For normally distributed continuous data, ANOVA was used to compare among three groups and independent t test to compare between each 2 groups. For non-normally distributed continuous data, Kruskal Wallis test was used to compare among three groups and Mann–Whitney U test to compare between each 2 groups. Chi-square or Fisher’s exact test was used to analyze categorical data (sex, ASA, surgeon, and adverse events). *P* values less than 0.05 were considered statistically significant.

## Results

Ninety-nine consecutive patients from March 2011 to July 2013 were enrolled in the study. Three patients were excluded due to requiring a fusion of more than three levels, leaving a total of 96 patients who were randomized: 32 into the group given ketorolac, 32 into the group given parecoxib, and 32 into the placebo group. There were no patients lost to follow-up and no patients were moved from one group into another group during the study. Also none of the patients withdrew from the study because of severe pain requiring additional analgesics beyond the intravenous morphine. The patient gender distribution was 33 males and 63 females. Thirty-six patients underwent surgery at one-level, 43 patients underwent surgery at two-levels and 17 patients underwent surgery at three-levels. There were no significant differences among the groups regarding gender, age, height, weight, ASA classification, operative level, operative time, blood loss during surgery, or the amounts of intraoperative narcotic (Table 1).

**Table 1:** **Patient characteristics and statistical analysis**

**Characteristics**	**Control**	**Ketorolac**	**Parecoxib**	***P***
**Number**	32	32	32	
**Sex**				0.575
**Male**	13 (40.6)	9 (28.1)	11 (34.4)	
**Female**	19 (59.4)	23 (71.9)	21 (65.6)	
Age (yr)*	55.6 ± 14	58.2 ± 9.5	58 ± 8.6	0.582
Weight (kg)*	66.5 ± 11	65.6 ± 12.2	64.3 ± 13.1	0.76
Height (cm)*	160.2 ± 8.1	159.5 ± 7.9	158.8 ± 7.7	0.776
BMI*	26 ± 4.8	26.4 ± 3.2	26 ± 3.6	0.913
**ASA status**				0.715
**I**	10 (31.25)	9 (28.13)	11 (34.37)	
**II**	22 (68.75)	23 (71.87)	21 (65.63)	
**Number fusion levels**				0.703
**I**	10 (31.2)	15 (46.9)	11 (34.4)	
**II**	15 (46.9)	13 (40.6)	15 (46.9)	
**III**	7 (21.9)	4 (12.5)	6 (18.8)	
Duration of surgery (min)*	165.7 ± 46.7	157 ± 33.3	189.2 ± 49.3	0.069
**Estimated blood loss (mL)**	450 (328.5)	489 (316.3)	587.5 (361.2)	0.246
**Intraoperative fentanyl**	162.6 (37.5)	157.4 (43.8)	175.9 (37.5)	0.121

*Abbreviations:*

BMI indicates body mass index.

ASA indicates American Society of Anesthesiologists.

*The values are given as mean and standard deviation.

### Verbal numerical rating score

**Figure 2:**
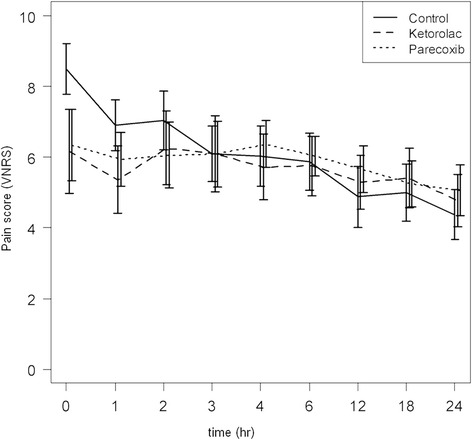
**Comparing pain intensity among the 3 groups during the first 24 hours after surgery.**

The wound pain scores of the patients as assessed by the VNRS after surgery showed that there was a statistically significantly average lower pain score reported at both 0 and 1 hours after surgery in the ketorolac group over the control group, and a statistically significantly average lower pain score at 0 hours after surgery in the group receiving parecoxib compared to the control group. However after repeated measurement by ANOVA test, there were no statistically significant differences between the parecoxib and ketorolac groups in pain reduction any time after surgery (Table 2) (Figure 2).

**Table 2:** **Pain intensity among the 3 groups during the first 24 hours after surgery**

**Time (hours)**	Pain scores*		
	**Control**	**Ketorolac**	**Parecoxib**
0	8.5(1.98)	6.1(3.30)	6.3(2.80)
1	6.9(2.00)	5.3(2.56)	5.9(2.09)
2	7.0(2.30)	6.2(2.85)	6.0(2.59)
3	6.0(2.16)	6.0(2.97)	6.0(2.58)
4	6.0(2.37)	5.7(2.58)	6.3(1.84)
6	5.8(2.25)	5.7(2.34)	6.0(1.53)
12	4.8(2.40)	5.2(2.10)	5.6(1.84)
18	5.0(2.24)	5.4(2.34)	5.2(1.79)
24	4.3(1.97)	4.7(2.05)	5.0(1.99)

*The values are given as mean and standard deviation.

### Supplement analgesic demands

The amount of morphine consumption after surgery in all three groups was not statistically significantly different at any of the recorded times in the 24 hours after surgery (Table 3).

**Table 3:** **Postoperative morphine consumption**

**Post operative morphine consumption at each time point**				
**Postoperative hours (hr)**	**Morphine (mg)**			
	**Control**	**Ketorolac**	**Parecoxib**	***P***
**8**	5.5 ± 1.9	5.3 ± 2.6	5.2 ± 2.3	0.86
**16**	5.7 ± 3.2	5.8 ± 4.2	4.9 ± 2.7	0.53
**24**	5.1 ± 5.4	6.4 ± 7	4.9 ± 4.6	0.55
**Total**	14.9 ± 9.3	16.4 ± 12.3	14.8 ± 8.1	0.79

The values are given as mean and standard deviation.

### Drain output

The amount of drain output was recorded until 24 hours after surgery. (Table 4) Blood volumes did not differ significantly among the three groups up to 24 hours after surgery.

**Table 4:** **Drain output**

**Drain output at each time point**				
**Post operative hour (hr)**	**Drain output (ml)**			
	**Control**	**Ketorolac**	**Parecoxib**	***P***
**0**	122.7 ± 89.2	107.9 ± 46.3	105 ± 51.2	0.514
**8**	105.9 ± 49.8	86.6 ± 66.6	114.5 ± 88.9	0.304
**16**	111.1 ± 87.6	76.2 ± 66.9	81.1 ± 61.1	0.122
**24**	108.3 ± 172.2	57.8 ± 99.4	84.4 ± 127.7	0.338
**Total**	408.1 ± 240.7	325.2 ± 204.6	373.7 ± 244.5	0.356

The values are given as mean and standard deviation.

### Adverse effects

The side effects that occurred and were recorded are shown in Table 5. In the control group, 2 patients complained of dyspepsia and 10 patients experienced nausea/vomiting. In the ketorolac group, 2 patients complained of dyspepsia and 12 experienced nausea/vomiting. In the parecoxib group, no patient complained of dyspepsia but 11 patients experienced nausea/vomiting. There were no major complications such as infection, respiratory depression, or urinary retention. There were no statistically significant differences in reported adverse effects among the three groups.

**Table 5:** **Incidence of adverse events during 48 hours**

**Incidence of adverse events during 48 hours**				
**Adverse events**	**Control No. (%)**	**Ketorolac No. (%)**	**Parecoxib No. (%)**	***P***
Dyspepsia	2 (8.7)	2 (8.7)	0 (0)	0.541
Nausea/vomiting	10 (31.2)	12 (37.5)	11 (34.4)	0.871
Constipation	2 (6.2)	1 (3.1)	2 (6.2)	1
Dizziness	5 (15.6)	8 (25)	6 (18.8)	0.632
Pruritus	2 (6.2)	2 (6.2)	5 (15.6)	0.496

## Discussion

Preemptive analgesia on experimental animal studies has shown central nervous system plasticity and sensitization after nociceptive stimulation [34]. Preemptive analgesia is defined as an anti-nociceptive treatment that prevents the establishment of altered central processing of afferent input which amplifies postoperative pain [35]. Administering an analgesic drug before pain stimulus can prevent the development of pain hypersensitization. However the concept that preemptive analgesia is more effective than conventional regimens in managing acute postoperative pain remains controversial [32]. There are many available analgesic interventions for possible preemptive analgesia effects in lumbar spinal surgery including epidural analgesia [36], local anesthetic wound infiltration [37], systemic opioids [38] and systemic NSAID drugs [39].

Postoperative pain following lumbar spinal fusion surgery can be perceived by the patient immediately in the recovery room as they are recovering after general anesthesia. Pain management in the postanesthesia care unit (PACU) is very important and at least one study has suggested that better management of pain in the PACU setting would likely improve patient satisfaction and facilitate shorter PACU stays [40]. Although there have been some studies evaluating the efficacy of NSAIDs for preemptive analgesia, our study is the first head-to-head study comparing ketorolac, parecoxib, and placebo for major orthopaedic surgery. We found that preemptive administration of parecoxib and ketorolac resulted in improved immediate postoperative pain at the PACU (0 and 1 hours in the ketorolac group and 0 hours in the parecoxib group). However, concerning the opioid-sparing analgesic effects, the amounts of morphine consumption were not different among the three groups, and also all three groups had comparable adverse events. So in this study, neither a reduction in opioid-type side effects nor opioid-sparing analgesic effects were demonstrated with the administration of single-dose preemptive parecoxib or ketorolac. These results may be explained because we gave only a single dose of parecoxib and ketorolac in this study.

Traditional NSAIDs are not recommended as a first choice supplemental analgesic in the perioperative setting due to the increased risk of perioperative bleeding [41,42]. But our study showed no differences between the 3 groups in terms of estimated blood loss and drain output in the first 24 hours after surgery, a finding which is comparable to a study by Ezequiel et al. [16] which also found no significant difference in postoperative drain output between their placebo and ketorolac groups.

Some studies have found an association between NSAID use and bone osteogenesis, which has important clinical implications for patients undergoing spinal fusion surgery [43,44]. Recent studies have found that a normal dose of an NSAID (<120 mg/d) did not appear to produce inferior results to a no-NSAIDs group in adult spinal fusion [45-47].

The only head-to-head comparison between parecoxib and ketorolac as a preemptive intravenous analgesia was in a study by Ng A. et al. [30] which compared the efficacy of intravenous parecoxib (40 mg) and intravenous ketorolac (30 mg) at induction. The study involved patients undergoing laparoscopic sterilization that is a short procedure. They concluded that parecoxib 40 mg at induction of anesthesia was less effective than ketorolac 30 mg in the first hour after laparoscopic sterilization. Our study was different than the Ng A. et al. study because it involved patients undergoing lumbar spinal fusion, which is a long, major orthopaedic operation. However, our study reached a comparable conclusion that both parecoxib and ketorolac are effective in reducing immediate acute postoperative pain in the recovery room, although the ketorolac seemed to take effect postoperatively one hour longer than parecoxib in our study. Although ketorolac has a shorter duration compared with parecoxib (6 hours from ketorolac and 12 hours from parecoxib), this result was different from a study by Romsing and Moiniche [20] that reported parecoxib 40 mg provided analgesic efficacy comparable to that of ketorolac with a longer duration of action after dental surgery but not after more extensive procedures. This might be explained by the pharmacokinetics of the drugs. Parecoxib is a prodrug which is metabolized in the liver to the active form called valdecoxib. After parecoxib 50 mg i.v., a Cmax of 1.02 mg/liter of the valdecoxib is achieved after 0.6 hours [48]. In contrast, ketorolac is administered in its active form and act immediately for COX inhibition. Through this type of mechanism, ketorolac could have a longer preemptive analgesia effect than parecoxib at one hour after surgery.

### Strengths and limitations

This is the first study to compare parecoxib, ketorolac, and placebo in posterior lumbar fusion surgery in the same setting in the same time period, following the CONSORT recommendations to provide increased confidence with unbiased results in estimating the effectiveness of an intervention [33].

This approach showed us the efficacy of both single-dose parecoxib and ketorolac in preemptive analgesia. Our calculated sample size had adequate power to detect key subgroup effects.

The limitation of this study is that we studied only single-dose ketorolac and parecoxib. Further studies, especially on the analgesic efficacy of repeated doses of parecoxib and ketorolac in surgical procedures are required.

## Conclusions

Preemptive analgesia using either ketorolac or parecoxib showed significantly better early postoperative pain control in the postanesthesia care unit (PACU) than the control group in patients undergoing lumbar spinal fusion.

## Abbreviations

NSAID: Nonsteroidal anti-inflammatory drug
PACU: Postanesthesia care unit
COX-2: Cyclo-oxygenase2 enzyme
CONSORT: Consolidated Standards of Reporting Trials
ASA: American Society of Anesthesiologists
VNRS: Verbal numerical rating score
ANOVA: Analysis of variance
